# Knockdown of HOXD13 in Oral Squamous Cell Carcinoma Inhibited its Proliferation, Migration, and Influenced Fatty Acid Metabolism

**DOI:** 10.7150/jca.102100

**Published:** 2025-01-01

**Authors:** Xingyue Ma, Xiao Zhang, Haiyang Li, Shuang Mei, Bowen Wang, Shuai Guan, Yitong Wang, Yuantao Li, Siyi Li, Xiangjun Li

**Affiliations:** 1 Department of Oral and Maxillofacial Surgery, School of Stomatology, Hebei Medical University, Hebei Technology Innovation Center of Oral Health, Key Laboratory of Stomatology and Clinical Research Centre for Oral Diseases, Hebei Province, Shijiazhuang, 050017, China.; 2 Department of Oral & Maxillofacial-Head & Neck Oncology, Shanghai Ninth People's Hospital, Shanghai Jiao Tong University School of Medicine, College of Stomatology, Shanghai Jiao Tong University, National Center for Stomatology, National Clinical Research Center for Oral Diseases, Shanghai Key Laboratory of Stomatology, Shanghai, 200011, China.

**Keywords:** OSCC, HOXD13, bioinformatics, TCGA, metabolism

## Abstract

**Background:** HOXD13, a member of the homeobox gene family, plays a critical role in developmental processes and has been implicated in various malignancies, including pancreatic cancer and glioma. However, its role in oral squamous cell carcinoma (OSCC) remains poorly understood. This study aimed to elucidate the potential of HOXD13 as a diagnostic biomarker and therapeutic target for OSCC.

**Methods:** We conducted a comprehensive analysis of OSCC samples from the Gene Expression Omnibus (GEO) and The Cancer Genome Atlas (TCGA)-head and neck squamous cell carcinoma (HNSCC) databases. Differentially expressed genes (DEGs) with upregulated expression were identified using Venn diagrams. Functional annotation was performed using Gene Ontology (GO) and Kyoto Encyclopedia of Genes and Genomes (KEGG) pathway enrichment analyses. A protein-protein interaction (PPI) network was constructed, and 10 key hub genes were identified using the cytoNCA method in Cytoscape. Subsequently, these hub genes were validated using quantitative real-time PCR (qRT-PCR) in tissue samples and cell lines. The impact of HOXD13 knockdown on OSCC cell proliferation and migration was assessed through lentiviral transduction followed by Cell Counting Kit-8 (CCk-8), 5-Ethynyl-2'-deoxyuridine (EdU), wound healing, and Transwell assays. Additionally, proteomic sequencing was performed to explore the effects on lipid metabolism-related pathways.

**Result:** Bioinformatic analysis revealed 121 upregulated DEGs in OSCC. Among these, 10 hub genes (FOXM1, CSF2, FN1, HOXD13, MMP9, SPP1, BIRC5, CXCL11, CXCL9, and FOXA2) were identified using the PPI network and Cytoscape analysis. HOXD13 was notably upregulated in OSCC tissues and cell lines, showing high diagnostic potential with an area under the receiver operating characteristics (ROC) curve (AUC) of 0.9. Immune infiltration analysis indicated significant differences in the tumor microenvironment associated with HOXD13 expression levels. Stable knockdown of HOXD13 in OSCC cell lines resulted in a marked reduction in cell proliferation and migration. Proteomic analysis post-HOXD13 knockdown highlighted alterations in fatty acid degradation pathways and increased expression of related metabolic enzymes.

**Conclusion:** HOXD13 is significantly upregulated in OSCC, and its inhibition reduces OSCC cell proliferation and migration. Additionally, HOXD13 affects fatty acid metabolism in OSCC, suggesting its potential as a therapeutic target and biomarker.

## Introduction

Oral cancer is a common malignancy with increasing incidence worldwide, significantly affecting patients' quality of life 1. Established risk factors for oral cancer include betel nut chewing, alcohol consumption, tobacco smoking, and human papillomavirus (HPV) infection 2. Over 90% of oral cancers are oral squamous cell carcinomas (OSCC) 1, which are frequently diagnosed at advanced stages due to inadequate early detection. Current primary treatment options include surgical resection, radiation therapy, and chemotherapy 2. Early-stage OSCC is often asymptomatic, leading to delayed diagnosis and reduced survival rates. Research estimates a 5-year survival rate of approximately 50% for patients with OSCC 3. Although histopathological examination is the diagnostic gold standard, it remains invasive and complex, so more refined diagnostic methods are needed 4.

HOX genes, members of the homeobox gene superfamily, play critical roles in various biological processes, including apoptosis, receptor signaling, and angiogenesis 5. Four distinct HOX gene clusters have been identified: HOXA, HOXB, HOXC, and HOXD, with HOXD located on chromosome 2q31 6. Germline mutations in HOXD13 are linked to congenital limb malformations 7, and amplification of alanine repeats in HOXD13 is associated with polydactyly 8. Moreover, HOXD13 acts as a rheostat for EWS::FLI1 transcriptional activity in Ewing sarcoma. Thus, its downregulation can alter the developmental gene program and expression of EWS::FLI1 target genes. HOXD13 binding EWS::FLI1 binding sites can inhibit EWS::FLI1 activator genes and activate suppressor genes 9. Furthermore, miR-7156-3p expression is markedly decreased in gliomas compared to normal brain tissue. As a target of miR-7156-3p, HOXD13 regulates stem cell proliferation and glioma cell aggressiveness 10. Despite HOXD13 being implicated in various cancers, its role in OSCC has not been previously reported. This study aimed to elucidate the potential of HOXD13 as a diagnostic biomarker and therapeutic target for OSCC. We hypothesized that elevated levels of HOXD13 are present in OSCC tissues and correlate with poor prognosis. Additionally, we posit that high HOXD13 expression is associated with immune cell infiltration in OSCC and is involved in lipid metabolism, impacting OSCC cell proliferation and migration.

## Materials and Methods

### Data acquisition

OSCC raw data were retrieved from The Cancer Genome Atlas (TCGA) database (https://portal.gdc.cancer.gov/), excluding non-oral head and neck squamous cell carcinoma (HNSCC) sites such as hypopharynx, larynx, lip, tonsils, and oropharynx in April 2023. A total of 339 OSCC and 32 normal tissue samples were selected, and their RNA-Seq transcriptomic and clinical data were obtained. Additional OSCC gene expression profiling data were acquired from the Gene Expression Omnibus (GEO) database (https://www.ncbi.nlm.nih.gov/geo/), 32 samples were collected from GSE23558 database, including 27 OSCC samples and 5 control samples, 80 samples were collected from GSE37991 database, including 40 OSCC samples and 40 control samples.

### Identification of Differentially Expressed Genes

Differentially expressed genes (DEGs) were identified by analyzing the TCGA-OSCC, GSE23558, and GSE37991 databases using |log_2_FC| ≥ 1.5 and Padj < 0.05 as thresholds. Upregulated DEGs common to all three databases were selected, as detailed in Table S1.

### Functional enrichment analysis

Functional enrichment analysis of upregulated DEGs was performed using the R package "clusterProfiler" for Gene Ontology (GO) and Kyoto Encyclopedia of Genes and Genomes (KEGG) pathway analysis. GO analysis included cellular components (CC), biological processes (BP), and molecular functions (MF). Results were visualized using the R package "ggplot2" with a threshold of Padj < 0.001, count ≥ 3, and the top four results presented.

### Construction of Protein-Protein Interaction (PPI) network

A PPI network for upregulated genes was constructed using the STRING database (http://string-db.org) and imported into Cytoscape. The cytoNCA Betweenness Centrality (BC) algorithm was utilized to identify 10 upregulated hub proteins.

### Receiver Operating Characteristic (ROC) curve analysis

ROC curve analysis was employed to evaluate the diagnostic potential of the expression of the 10 relevant genes using the "pROC" software package to calculate the area under the curve (AUC). Sensitivity and specificity for disease prediction were also assessed.

### Correlation analysis of immune infiltration of hub genes

HOXD13 expression levels in TCGA-OSCC were categorized into high- and low-expression groups based on the median cutoff value. DEGs were identified using the DESeq2 package in R, and volcano plots were generated with |log2FC| > 1 and Padj < 0.05. The correlation between HOXD13 expression and immune cell infiltration in OSCC was analyzed using the Single-sample Gene Set Enrichment Analysis (ssGSEA) algorithm from the R package GSVA (version 1.46.0) 11 and markers for 24 types of immune cells from the Immunity Article 12. These markers were employed to determine the relative proportions of tumor-infiltrating immune cells in each sample.

### GSEA enrichment analysis

The "GSEA" package in R software was used to investigate the role of HOXD13 in OSCC. Enrichment analysis was conducted using the gene set "c2.cp.kegg.v7.4.symbols.gmt" from the GSEA MSigDB database (https://www.gsea-msigdb.org/gsea/msigdb), with selected gene sets displayed.

### Tissue collection

Tissue samples from 18 patients diagnosed with OSCC at the Stomatological Hospital of Hebei Medical University and Cangzhou Central Hospital between October 2022 and August 2024 were collected. The gingival mucosal epithelium of 10 impacted teeth that were extracted was utilized as the control group. Informed consent was obtained from all patients, and the study was conducted in accordance with the Declaration of Helsinki. This study was approved by the Medical Ethics Committee of Hebei Province. Patient demographic characteristics are summarized in Table S2.

### Cell culture

OSCC cell lines SCC9, SCC25, and CAL27 were obtained from Procell Biology (Wuhan, China); SCC4 from Mingjin Biology (Shanghai, China); and the normal oral epithelial cell line (HOK) from Otwo Biotech (Guangzhou, China). SCC9, SCC4, and SCC25 cells were cultured in DMEM/F12 supplemented with hydrocortisone (Invitrogen). HOK and CAL27 cells were cultured in DMEM medium (Gibco) with 10% fetal bovine serum (Gibco), 1% penicillin (100 U/mL), and streptomycin (100 μg/mL) in a 37 °C incubator with 5% CO_2_.

### Quantitative Reverse Transcription PCR (qRT-PCR)

Total RNA was extracted using the R0026 kit (Beyotime, China) and reverse-transcribed into complementary DNA (cDNA) with the PrimeScript RT kit (Takara, Japan). Real-time quantitative PCR was performed using an SYBR premixed Ex Taq kit (Takara, Japan) and an ABIQ5 real-time PCR system (Applied Biosystems, USA). Relative mRNA expression was calculated using the 2^-ΔΔCT^ method. Primer sequences were: HOXD13 forward primer: 5ʹ-CGGCGTATCTCGGCTGCTAC-3ʹ, reverse primer: 5ʹ-TCTTGTCCTTCACTCTTCGGTTCTG-3ʹ; GAPDH forward primer: 5ʹ-CAGGAGGCATTGCTGATGAT-3ʹ, reverse primer: 5ʹ-GAAGGCTGGGGCTCATTT-3ʹ.

### Western blotting analysis

Total protein was extracted using RIPA buffer and PMSF (Soleibao, China), with protein concentration determined using a BCA protein detection kit (Soleibao, China). Proteins were separated by electrophoresis and transferred to PVDF membranes (Millipore, Boston, MA, USA). Membranes were blocked with protein-free rapid blocking solution (Yadase, China), incubated with primary antibodies overnight at 4 °C, and then incubated with secondary antibodies. After washing with TBST, protein expression levels were quantified using ImageJ software. Antibodies used included HOXD13 (Proteintech No.18736-1-AP), β-tubulin (Proteintech No: CL488-66240), CPT2 (Abways CY5699), ACOX3 (HUABIO ER60440), ACADVL (Abways CY8651), ECHS1 (Proteintech, No. 66117-1-Ig), HADHA (Proteintech, No. 60250-1-Ig), and GAPDH (Abways, AB2000).

### RNA knockdown vector construction and proteomic sequencing analysis

An shRNA lentiviral vector targeting HOXD13 was designed and synthesized (Gemma Gene, China). CAL-27 and SCC-9 cells were seeded in 6-well plates overnight and transfected with the lentivirus 24 h later. RNA and protein were extracted 72 and 96 h post-transfection, respectively, for RT-qPCR and western blotting to assess gene knockdown efficiency. sh-HOXD13-1: GCTCTAAATCAGCCGGACATG; sh-HOXD13-2: GGAGAACGAGTATGCCATTAA; sh-HOXD13-3: GAGAGACAAGTGACCATTTGG. The sh-NC and sh-HOXD13-2 groups in CAL27 cells were collected, with three samples per group, each containing 1×10^7^ cells, for proteomic analysis.

### Cell Counting Kit-8 (CCK-8) assays

Cells transfected with lentivirus for 72 h were seeded in 96-well plates at a density of 2000 cells/well in triplicate. After adding 10 μL of CCK-8 reagent to 90 μL medium (China, pottery), cells were incubated at 37°C for 2 h. Absorbance at 450 nm was measured using a Bio-Tek spectrophotometer over 1-5 days.

### 5-Ethynyl-2'-deoxyuridine (EdU) assays

EdU kits were procured from Beyotime Biotechnology (China). Cells (2×10^5^) were plated in 6-well plates, treated with 1× EdU working solution, fixed at room temperature for 15 min, washed with washing solution, and permeabilized for 10-15 min at room temperature. A click reaction solution was prepared and incubated at room temperature for 30 min in the dark. After three washes, Hoechst 33342, a blue fluorescent dye, was used for nuclear staining, with fluorescence detected at an excitation wavelength of 346 nm and an emission wavelength of 460 nm.

### Wound healing assays

Cells were seeded in 24-well plates at a density of 300,000 cells per well. Once confluent, a scratch was created using a pipette tip. Images of cell migration into the wound area were captured at 0, 12, 24, and 36 h using Cellomics (ArrayScan VT1; ThermoFisher Scientific) and analyzed with ImageJ software.

### Transwell Assays

Cells were seeded into transwell chambers at a density of 60,000 cells per well. A serum-free cell suspension was added to the upper chamber, and 20% FBS + DMEM culture medium was added to the lower chamber. After 48 h, non-migrating cells were removed with a cotton swab, stained with crystal violet for 20 min, and imaged under a fluorescence microscope. The number of migrated cells was quantified.

### Statistical Analysis

Statistical analysis was performed using GraphPad Prism 9 software (GraphPad, La Jolla, CA, USA). Comparisons were made using Student's t-test or one-way analysis of variance (ANOVA), with statistical significance set at P < 0.05.

## Results

### Identification of key upregulated genes and ROC curve analysis

**Figure 1:**
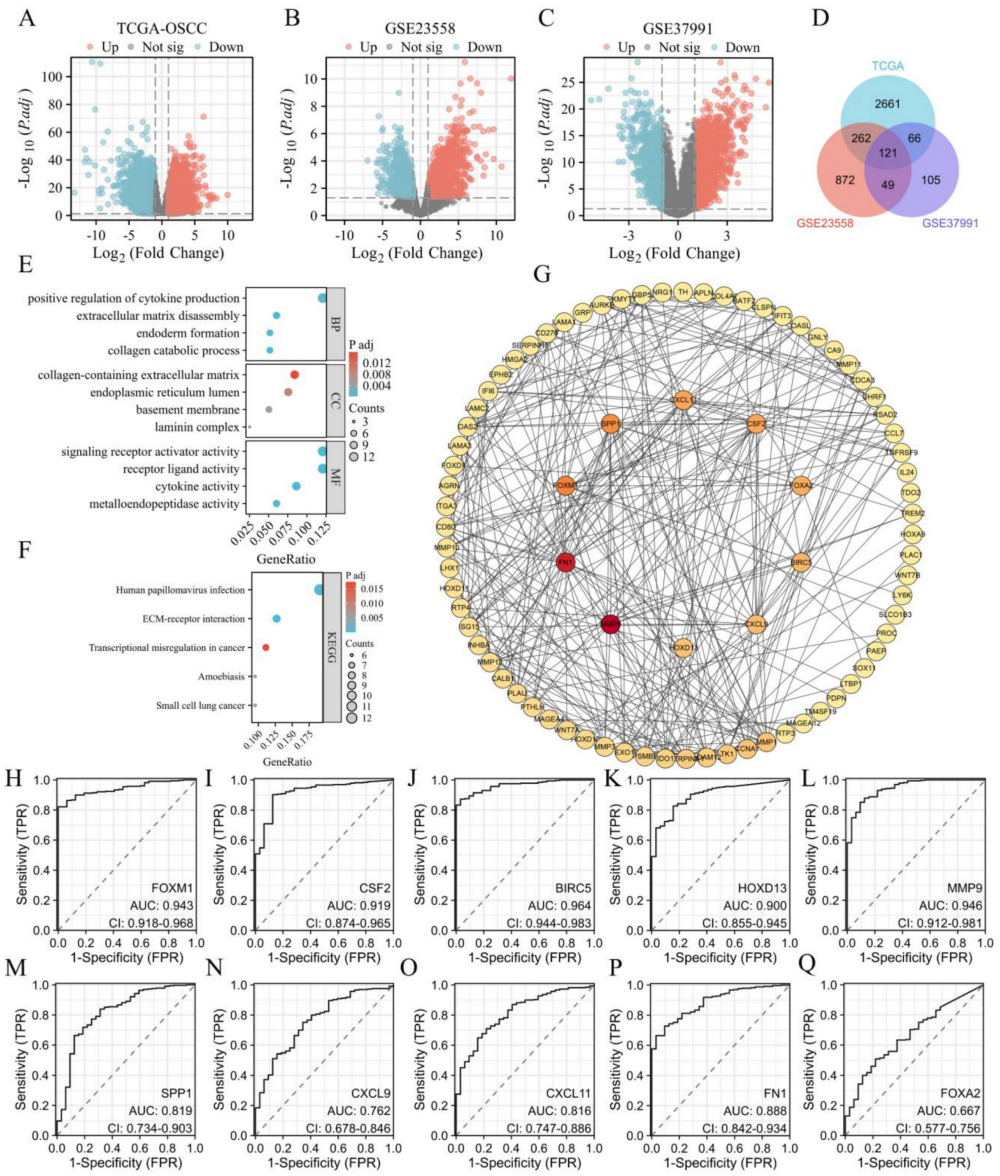
A: Volcano plot showing differentially expressed genes (DEGs) in TCGA-OSCC. Upregulated genes are indicated in red, and downregulated genes are in cyan. B: Volcanic plot of DEGs in GSE23558 with similar color coding for upregulated (red) and downregulated (cyan) genes. C: Volcano plot of DEGs in GSE37991, with upregulated genes highlighted in red and downregulated genes in cyan. D: Venn diagram illustrating the intersection of 121 upregulated DEGs from TCGA, GSE23558, and GSE37991 datasets. E: Gene Ontology (GO) enrichment analysis of the 121 upregulated DEGs. F: KEGG pathway enrichment analysis of the 121 upregulated DEGs. G: Identification of top 10 hub genes. H-Q: Receiver Operating Characteristic (ROC) curves and Area Under the Curve (AUC) values for FOXM1, CSF2, BIRC5, HOXD13, MMP9, SPP1, CXCL9, CXCL11, FN1, and FOXA2.

In this study, we focused on identifying and analyzing upregulated DEGs in OSCCs. Analysis of the TCGA database identified 3110 upregulated DEGs, while the GSE23558 and GSE37991 databases revealed 1304 and 341 upregulated DEGs, respectively (Figures 1A-C). The intersection of these upregulated genes across all three databases resulted in 121 common DEGs (Figure 1D). Functional enrichment analysis through GO and KEGG revealed that these DEGs were significantly involved in the positive regulation of cytokine production, extracellular matrix (ECM) remodeling, endoderm formation, and collagen catabolic processes in the BP category. In the CC category, DEGs were associated with the ECM, endoplasmic reticulum lumen, basement membrane, and laminin complex. MF analysis indicated associations with receptor signaling, ligand-receptor interactions, and cytokine activities. KEGG pathway analysis highlighted the enrichment of pathways related to HPV infection, ECM-receptor interactions, and transcriptional dysregulation in cancer (Figure 1F). The 121 upregulated genes were further analyzed using the STRING database to construct a PPI network and visualized using Cytoscape software. The BC algorithm identified the top 10 hub genes (Figure 1G), which exhibited strong interactions with each other. ROC curve analysis showed that all 10 hub genes had an AUC > 0.6, with FOXM1, CSF2, BIRC5, HOXD13, and MMP9 displaying AUC values ≥ 0.9 (Figures 1H-Q).

### Validation of hub gene at tissue and cellular levels

**Figure 2:**
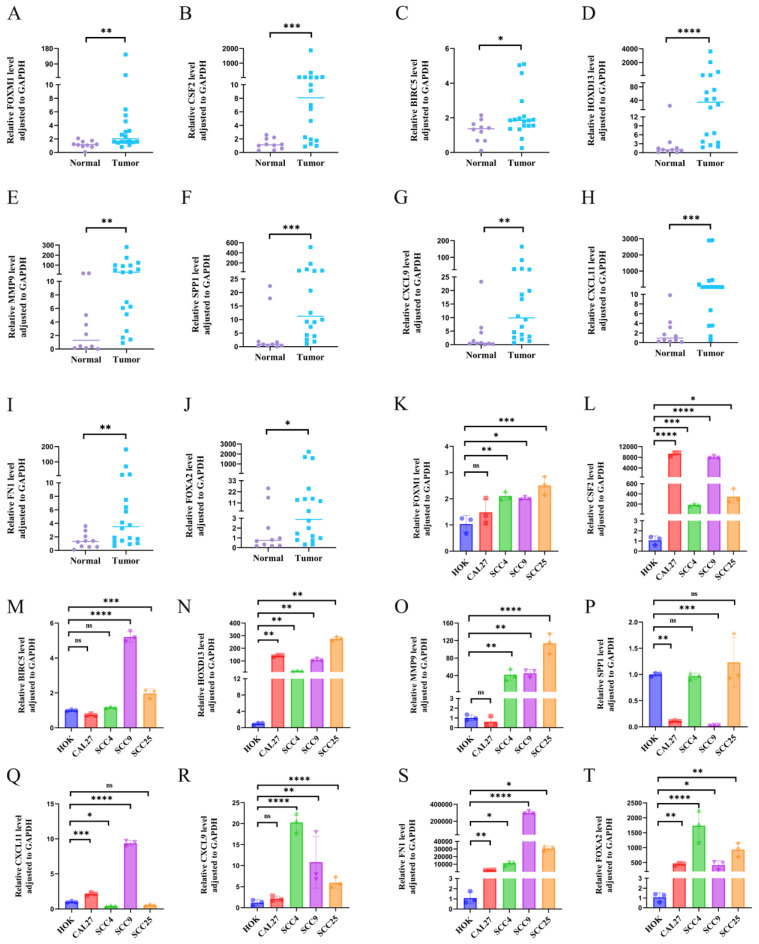
A-J: RT-qPCR analysis showing tissue expression levels of FOXM1, CSF2, BIRC5, HOXD13, MMP9, SPP1, CXCL9, CXCL11, FN1 and FOXA2. K-T: Gene expression validation in four OSCC cell lines (CAL-27, SCC-4, SCC-9, SCC-25) and the normal oral epithelial cell line HOK, using RT-qPCR. GAPDH was used as an internal reference gene. Data are shown as mean ± standard deviation (SD), with statistical analysis performed using a two-tailed unpaired t-test (n = 3 independent experiments). (*P < 0.05, **P < 0.01, ***P < 0.001).

The expression levels of the 10 hub genes were validated using RT-qPCR on samples from18 OSCC tissues and 10 normal controls. All 10 hub genes were significantly upregulated in OSCC tissues compared to normal tissues (Figures 2A-J). Validation at the cellular level using OSCC and HOK cell lines revealed that CSF2, FN1, HOXD13, and FOXA2 were significantly upregulated in OSCC cell lines (CAL-27, SCC-4, SCC-9, and SCC-25) compared to HOK cells, the expression of FOXM1, MMP9, SPP1, BIRC5, CXCL11, and CXCL9 was not so consistent in all OSCC cell lines (Figures 2K-T). Figures K, O, and R show that FOXM1, MMP9, and CXCL9 were not significantly different from HOK in CAL-27 cells. Figure P: The expression of SPP1 was not upregulated in the four tumor cell lines compared to that in the HOK cell line. Figure M: The expression of BIRC5 in CAL27 and SCC4 cell lines was not significantly different from that in the HOK cell line. Figure Q: CXCL11 showed no statistical significance in SCC25 cells relative to HOK cells.

### Analysis of HOXD13 expression and immune infiltration

**Figure 3:**
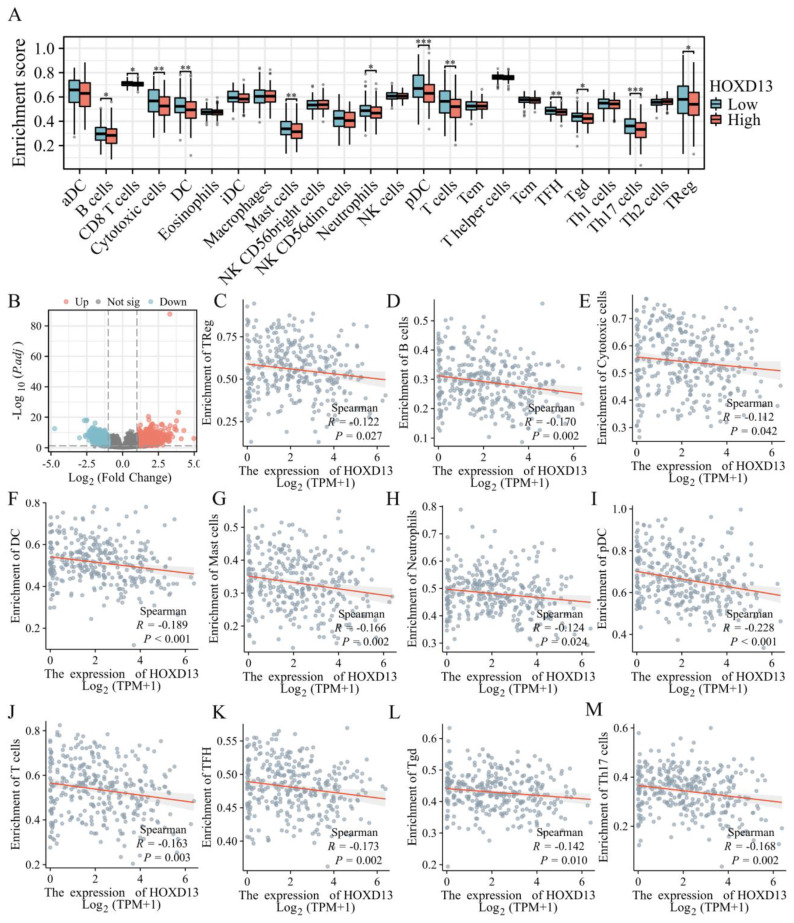
A: Correlation between HOXD13 expression levels and the relative abundance of 24 immune cell types; B: Volcano plot based on median HOXD13 expression levels in the TCGA dataset; C-M: Scatter plots showing the correlation between HOXD13 expression and various immune cell types.

To explore the role of HOXD13 in immune infiltration, patients were stratified into high and low HOXD13 expression groups based on median expression levels from the TCGA database. A volcano plot was used to visualize the expression differences (Figure 3B). A comparison of the enrichment scores for 24 immune cell types revealed higher scores for 12 immune cell types in the HOXD13 low-expression group (Figure 3A).

ssGSEA indicated a negative correlation between HOXD13 expression and various immune cells, including regulatory T cells (Treg) (R = -0.122), B cells (R = -1.70), cytotoxic cells (R = -0.112), dendritic cells (DC) (R = -0.189), mast cells (R = -0.166), neutrophils (R = -0.124), plasmacytoid dendritic cells (pDC) (R = -0.228), T cells (R = -0.163), Follicular helper T cell(TFH)( R = -0.173), gamma delta T cells (Tgd) (R = -0.142), and Th17 cells (R = -0.168) (Figures 3C-M).

### Establishment of stable knockdown cell lines

**Figure 4:**
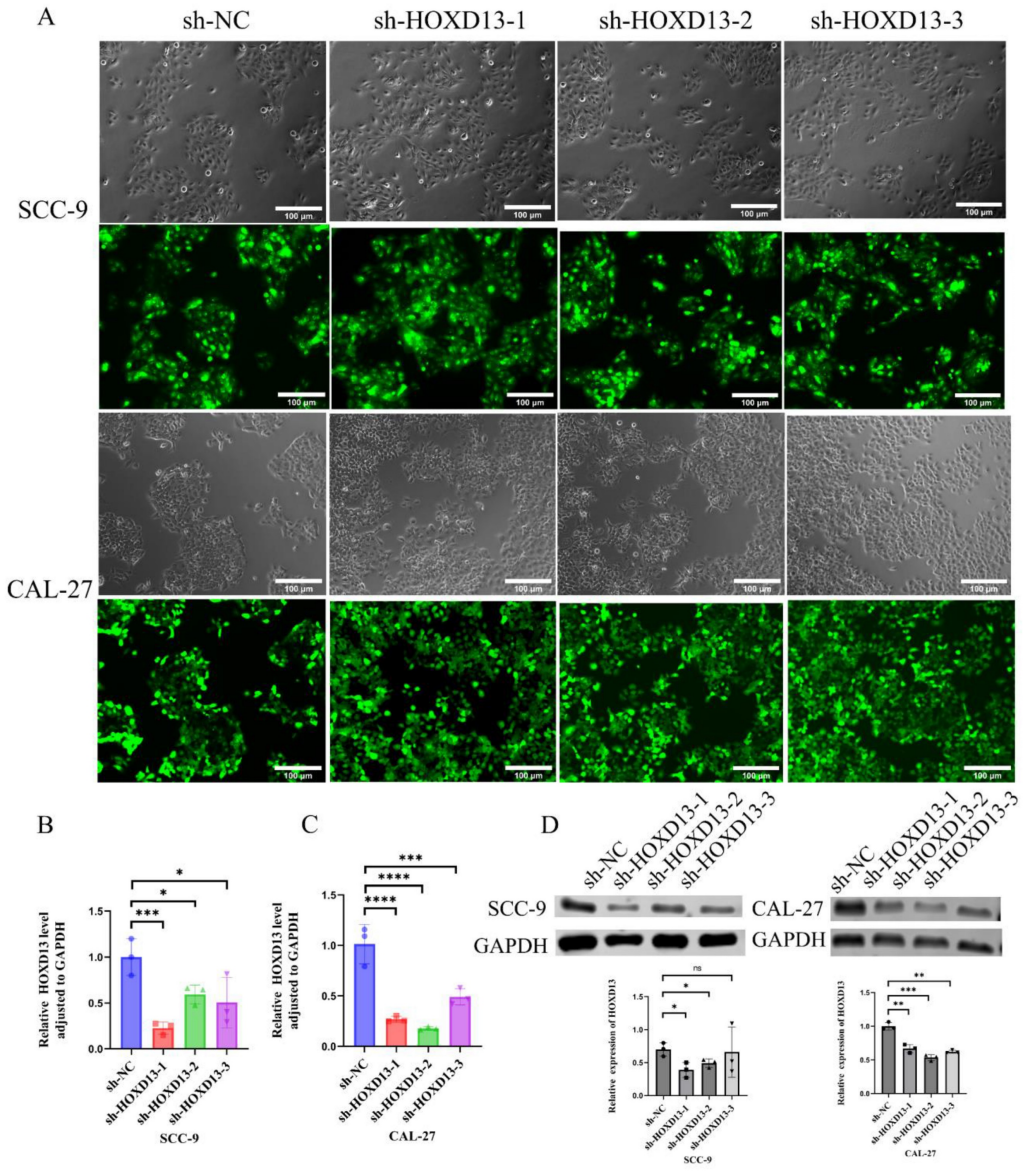
A: Lentiviral transduction of SCC-9 and CAL-27 cell lines with HOXD13 knockdown constructs. B-C: qRT-PCR analysis confirming HOXD13 expression levels in three knockdown groups versus control in SCC-9 and CAL-27 cell lines. D: Western blot analysis of HOXD13 protein expression in SCC-9 and CAL-27 cells. Data are expressed as mean ± standard deviation (SD), with statistical significance determined by two-tailed unpaired t-tests (n = 3 independent experiments). (* P < 0.05, **P < 0.01, ***P < 0.001).

Stable HOXD13 knockdown cell lines were generated using lentiviral vectors (Figure 4A). qRT-PCR confirmed significant reductions in HOXD13 expression in knockdown groups compared to controls (Figures 4B-C). Western blotting further confirmed the downregulation of HOXD13 protein levels in SCC-9 and CAL-27 cell lines (Figure 4D). These two knockdown groups were selected for subsequent functional assays.

### Impact of HOXD13 Knockdown on OSCC Cell Proliferation and Migration

**Figure 5:**
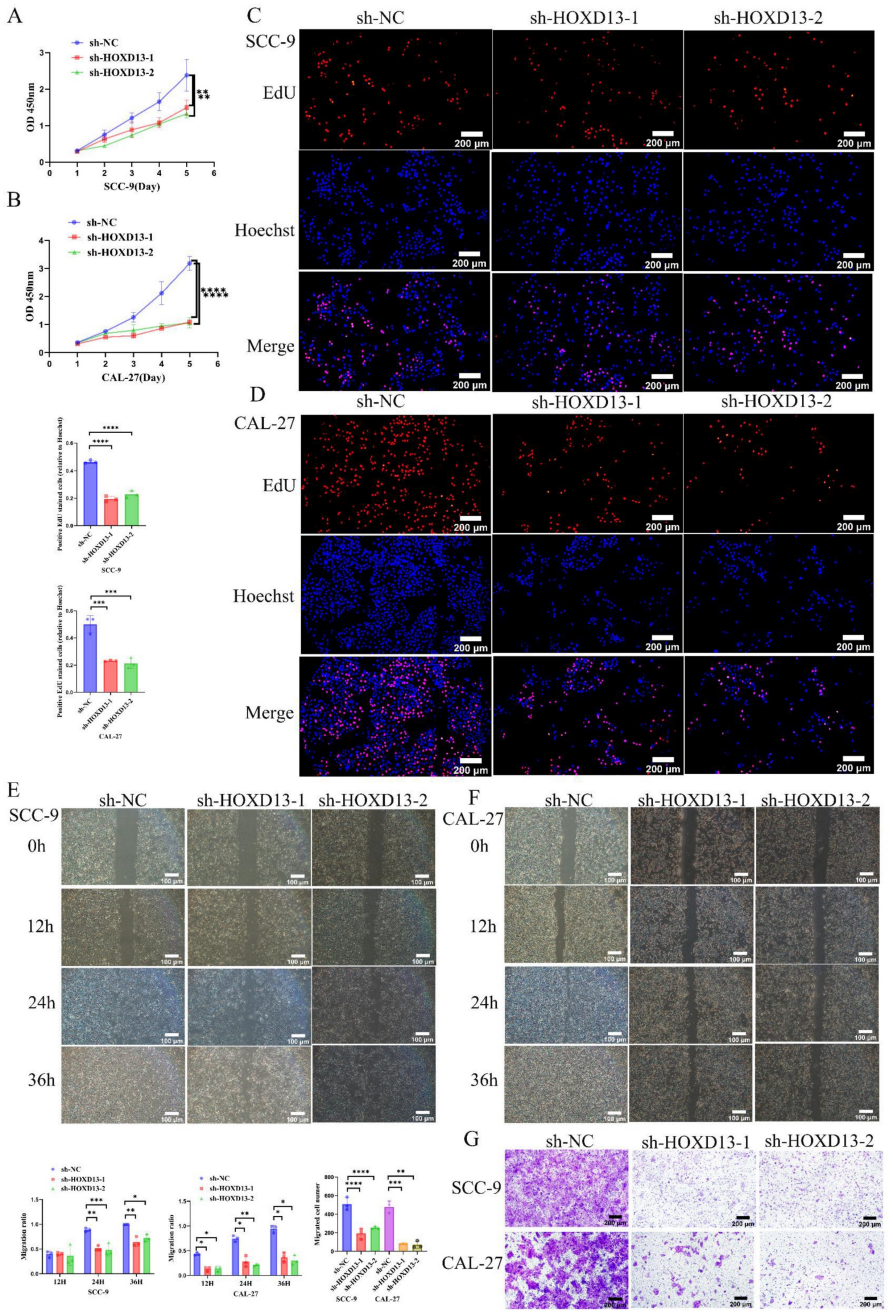
D: CCK-8 and EdU assays assessing cell proliferation in SCC-9 and CAL-27 cell lines with HOXD13 knockdown compared to control. E-G: wound healing assays and transwell migration tests evaluating cell migration ability in HOXD13 knockdown groups versus control in SCC-9 and CAL-27 cell lines. Results are presented as mean ± standard deviation (SD) with statistical analysis determined by two-tailed unpaired t-tests (n = 3 independent experiments). (*P < 0.05, **P < 0.01, ***P < 0.001).

The CCK-8 assay demonstrated that HOXD13 knockdown significantly inhibited OSCC cell proliferation in SCC-9 and CAL-27 cell lines, starting from day 3 (Figures 5A-B). EdU incorporation assays revealed over 50% inhibition of proliferation in HOXD13 knockdown groups compared to controls (Figures 5C-D), indicating that HOXD13 downregulation impairs OSCC cell proliferation.

Wound healing assays showed reduced healing rates at 24 h in SCC-9 cells and at 12 h in CAL-27 cells with HOXD13 knockdown (P < 0.05) (Figures 5E-F). Transwell assays confirmed that HOXD13 knockdown significantly inhibited cell migration in SCC-9 (P < 0.01) and CAL-27 (P < 0.0001) cells (Figure 5G), demonstrating that HOXD13 downregulation affects both proliferation and migration of OSCC cells.

### Effect of HOXD13 knockdown on fatty acid metabolism

**Figure 6:**
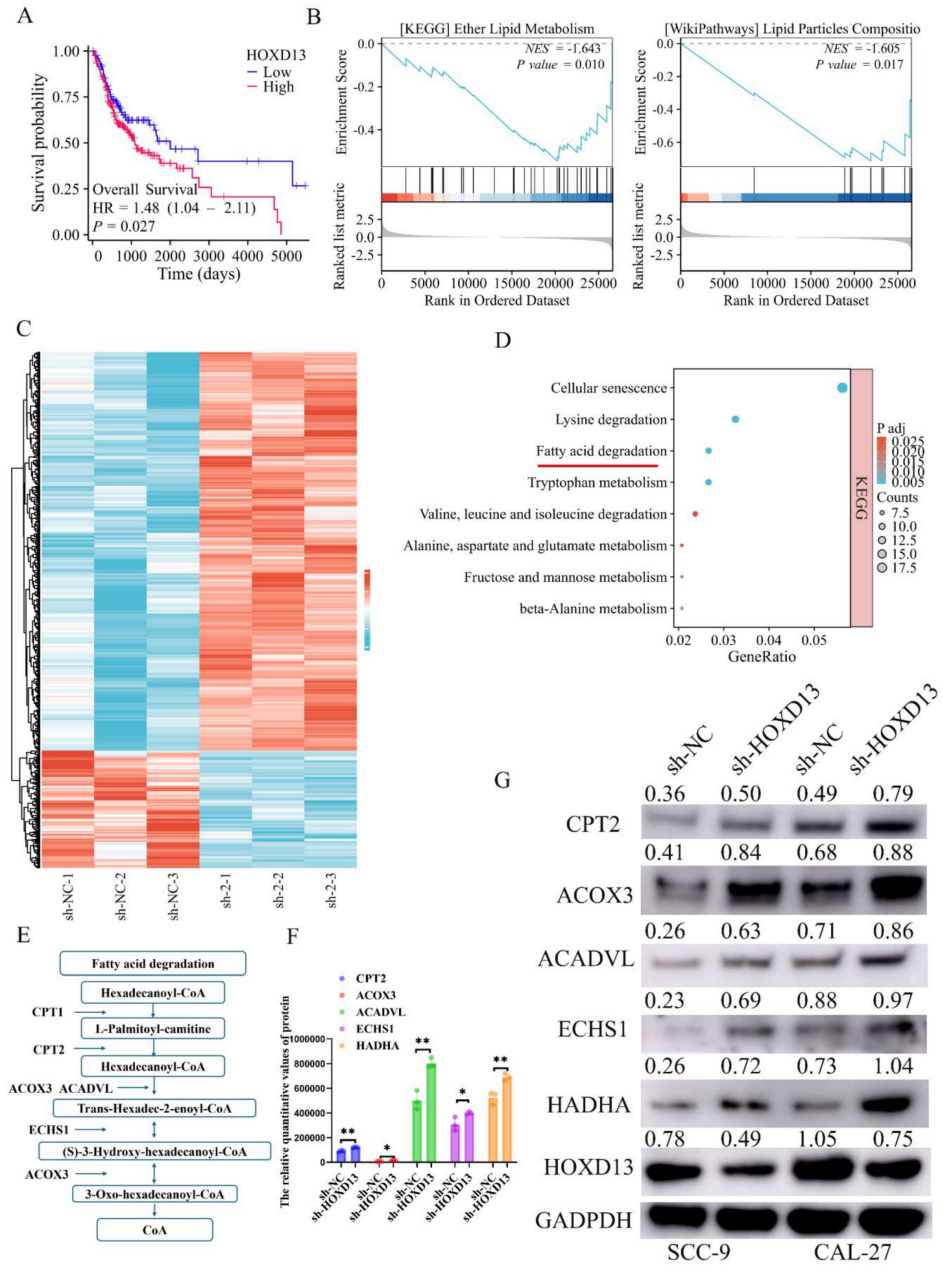
A: Prognostic analysis map of HOXD13 in TCGA dataset. B: Gene Set Enrichment Analysis (GSEA) results showing enrichment of lipid metabolism-related pathways in high versus low HOXD13 expression groups. C: Heatmap of proteomic sequencing data showing DEG expression in CAL-27 cells with HOXD13 knockdown compared to control. D-E: KEGG enrichment analysis of differentially expressed proteins, highlighting fatty acid degradation pathways. F: Protein expression levels of CPT2, ACOX3, ACADVL, ECHS1, and HADHA in CAL-27 cells post-HOXD13 knockdown. G: Validation of protein expression levels of CPT2, ACOX3, ACADVL, ECHS1, and HADHA in SCC-9 and CAL-27 cell lines.

Clinical data from the TCGA database indicated that high HOXD13 expression was associated with poor prognosis (Figure 6A). GSEA of HOXD13 high and low expression groups identified significant enrichment in lipid metabolism-related pathways (Figure 6B). Accordingly, the sh-NC and sh-HOXD13-2 (sh-2) groups of the CAL-27 cell line were subjected to proteomics sequencing. The results revealed 499 upregulated and 147 downregulated proteins, using |log2FC| ≥ 1.2 and P-value < 0.05 as screening criteria (Figure 6C). KEGG enrichment analysis of these differential proteins highlighted the fatty acid degradation pathway (Figures 6D, E). Proteomic analysis indicated increased protein expression of CPT2 ACOX3, ACADVL ECHS1, and HADHA following HOXD13 knockdown (Figure 6F), the results were verified by Western blot experiments (Figure 6G). These findings suggest that HOXD13 affects OSCC progression by influencing fatty acid metabolism.

## Discussion

OSCC is a prevalent malignancy of the head and neck region, characterized by a poor prognosis due to low 5-year survival rates and high rates of tumor recurrence and metastasis. To improve patient outcomes, identifying potential therapeutic targets for OSCC is crucial. In this study, bioinformatic analyses of OSCC samples from the TCGA and GEO (GSE23558 and GSE37991) databases led to the identification of 10 key genes-FOXM1, CSF2, FN1, HOXD13, MMP9, SPP1, BIRC5, CXCL11, CXCL9, and FOXA2-implicated in OSCC pathogenesis.

Among these genes, FOXM1 has been identified as a critical factor in OSCC, promoting tumor progression through mechanisms such as epithelial-mesenchymal transition (EMT) 13, 14. Thiostrepton, a specific inhibitor of FOXM1, has shown potential in overcoming radioresistance in OSCC cell lines, positioning it as a promising therapeutic agent 15. Additionally, glycogen synthase kinase-3β (GSK-3β) mediates OSCC progression and invasion by regulating MMP9 16, while the circRNA hsa-circ-0009128 modulates EMT through MMP9 expression, influencing OSCC cell proliferation and migration 17. Other genes, including CSF2 18, FN1 19, FOXA2 20, SPP1 21, and BIRC5 22, have been proposed as prognostic biomarkers in OSCC. CXCL11 and CXCL9 have been linked to OSCC cell proliferation and migration, with knockdown studies demonstrating their impact on CD274 and IDO1 expression 23, 24. Our RT-qPCR validation revealed that CSF2, FN1, HOXD13, and FOXA2 exhibited expression patterns consistent with our bioinformatics analyses. However, the validation and bioinformatics results of FOXM1, MMP9, SPP1, BIRC5, CXCL11 and CXCL9 in cell lines were inconsistent, although they were consistent in tissue samples. Notably, HOXD13, with an AUC value of 0.9, emerged as the most significant DEG, underscoring its potential as a diagnostic marker.

HOXD13 has been implicated in various cancers, including colon cancer, where its knockdown inhibits cell proliferation and invasion 25. In gastric cancer, GALNT10 interacts with HOXD13, modulating cell proliferation and migration 26. In prostate cancer, HOXD13 binds to the SMAD1 promoter, disrupting BMP4-induced EMT and reducing tumor cell invasiveness 27. Abnormal HOXD13 expression has also been observed in breast cancer, melanoma, cervical cancer, astrocytoma, and pancreatic cancer 28. Consistent with this report, this study confirms that HOXD13 is overexpressed in OSCC tissues and cells compared to normal controls and correlates with immune infiltration. Moreover, the knockdown of HOXD13 in OSCC cell lines resulted in significantly reduced cell proliferation and migration.

To elucidate the mechanisms by which HOXD13 influences OSCC, we performed GSEA and proteomic sequencing. Our findings suggest that HOXD13 may impact fatty acid metabolism in OSCC. Cancer cells often exhibit altered lipid metabolism, leading to metabolic dysfunction, a hallmark of cancer cells 29. Research indicates that fatty acids are essential for cell membrane formation, ATP, and NADPH production 30.

Specifically, it has been demonstrated that the disruption of fatty acid synthesis can induce apoptosis under lipid-depleted conditions 31, and cancer cells frequently enhance fatty acid metabolism to meet increased metabolic demands 32. In this study, GSEA revealed that HOXD13 expression correlates with lipid metabolism-related pathways. Furthermore, proteomic analysis showed that HOXD13 knockdown enriches the fatty acid degradation pathway and increases the expression of associated proteins, indicating that HOXD13 regulates fatty acid metabolism in OSCC.

One notable limitation of this study is the use of the BC algorithm in Cytoscape's CytoNCA method for identifying hub genes, which may differ from other methods, such as CytoHubba 33. Additionally, the selection of |logFC| ≥ 1.5 for gene screening, while effective, lacks universal guidelines. Future studies may benefit from exploring alternative algorithms and criteria for more precise results.

In conclusion, HOXD13 is highly expressed in OSCC and is associated with poor prognosis. It plays a role in immune infiltration and affects OSCC progression by modulating fatty acid metabolism pathways. HOXD13 holds promise as a valuable biomarker for the diagnosis and prognosis of OSCC and presents a potential therapeutic target for future interventions.

## Supplementary Material

Table S1**.** A list of 121 upregulated genes identified through intersection analysis of the three datasets. Table S2**.** Patients' demographic characteristics.
