# Supplementary material for: Knockdown of HOXD13 in Oral Squamous Cell Carcinoma Inhibited its Proliferation, Migration, and Influenced Fatty Acid Metabolism
Source: J Cancer. 2025 Jan 1;16(1):214–26. doi: 10.7150/jca.102100 (PMC11660140; doi:10.7150/jca.102100)
Supplement: Supplementary file 1 — Table S1. A list of 121 upregulated genes identified through intersection analysis of the three datasets. Table S2. Patients' demographic characteristics. [file jcav16p0214s1.pdf]

Supplementary Table 1

FN1	LAMA1	LAMA3	SUN3	IDO1	WNT7A
TDO2	AJAP1	TH	APLN	IFI6	HOXD11
SLC24A2	TK1	STX1A	CALB1	SERPINE1	HOXC13
NXPH4	MMP11	MMP12	CD80	GRP	RTP3
SPOCD1	LTBP1	TENM2	OASL	TM4SF19	BATF2
WNT7B	FADS3	EPHB2	TNFRSF9	ISG15	TREM2
BIRC5	LHX1	PAEP	COL4A6	IFIT3	INHBA
SERPINH1	C16orf74	ANXA13	SLC52A1	RSAD2	PTHLH
RTP4	EXO1	CCNA1	TMC7	CXCL9	CSF2
GNLY	HOXA9	TMEM92	SOX11	ZNF114	MISP
FXYD5	PSMB9	HOXD13	GPRIN1	IL1RL1	SLCO1B3
PYY2	UHRF1	EVA1A	PLAU	HMGA2	PLAC1
ADAM12	AGRN	FEZ1	KREMEN2	MAGEA12	TRIML2
ELAVL2	ALOX12P2	FOXM1	CCL7	FOLR3	SPP1
PTK7	HSD17B6	PKMYT1	FOXA2	GBP5	MMP1
MMP9	PROC	CLSPN	LAMC2	SCG5	CXCL11
PPEF1	CDCA3	NRG1	ITGA3	MAGEA4	HOXC9
FOXD1	CD276	OAS2	LY6K	PPP4R4	DHRS2
ARTN	AURKB	GNGT1	PDPN	CYP27B1	MMP10
SPRY4	HOXD10	HOXC8	IGFL2	IL24	MMP3
CA9					

Supplementary Table 2

tumor	age	gender	Sampling site	TNM	diagnosis	normal	age	gender	Sampling site
1	69	female	tongue	T2N0M0	Squamous cell carcinoma of the right tongue	1	18	male	gingiva
2	73	female	tongue	T1N0M0	Squamous cell carcinoma of the right tongue	2	16	male	gingiva
3	59	male	gingiva	T1N0M0	Carcinoma of the left lower gingiva	3	29	female	gingiva
4	34	male	cheek	T2N1M0	Squamous cell carcinoma of the left cheek	4	22	male	gingiva
5	53	male	tongue	T2N2bM0	Squamous cell carcinoma of the right tongue	5	23	female	gingiva
6	68	female	cheek	T2N0M0	Squamous cell carcinoma of the left cheek	6	23	male	gingiva
7	60	male	cheek	T2N0M0	Squamous cell carcinoma of the left cheek	7	28	male	gingiva
8	75	male	cheek	T2N0M0	Squamous cell carcinoma of the left cheek	8	27	female	gingiva
9	60	male	lip	T1N0M0	Squamous cell carcinoma of the right lower lip	9	17	female	gingiva
10	69	female	cheek	T2N2M0	Squamous cell carcinoma of the left cheek	10	22	male	gingiva
11	68	male	palate		Squamous cell carcinoma of the left palate				
12	71	male	gingiva		Carcinoma of the right lower gingiva				
13	62	male	tongue		Squamous cell carcinoma of the left tongue				
14	70	female	cheek		Squamous cell carcinoma of the left cheek				
15	78	female	cheek		Squamous cell carcinoma of the left cheek				
16	56	female	gingiva		Squamous cell carcinoma of the left upper gingiva				
17	72	male	cheek		Squamous cell carcinoma of the right cheek				
18	67	female	tongue		Squamous cell carcinoma of the left tongue				
